# Real-time intraoperative glioma diagnosis using fluorescence imaging and deep convolutional neural networks

**DOI:** 10.1007/s00259-021-05326-y

**Published:** 2021-04-27

**Authors:** Biluo Shen, Zhe Zhang, Xiaojing Shi, Caiguang Cao, Zeyu Zhang, Zhenhua Hu, Nan Ji, Jie Tian

**Affiliations:** 1 CAS Key Laboratory of Molecular Imaging, Beijing Key Laboratory of Molecular Imaging, The State Key Laboratory of Management and Control for Complex Systems, Institute of Automation, Chinese Academy of Sciences, 95 Zhongguancun East Road, Beijing, 100190 China; 2 School of Artificial Intelligence, University of Chinese Academy of Sciences, Beijing, China; 3 Department of Neurosurgery, Beijing Tiantan Hospital, Capital Medical University, No. 119 South Fourth Ring West Road, Fengtai District, Beijing, 100070 China; 4 China National Clinical Research Center for Neurological Diseases, Beijing, China; 5 Beijing Advanced Innovation Center for Big Data-Based Precision Medicine, School of Engineering Medicine, Beihang University, Beijing, China; 6 Engineering Research Center of Molecular and Neuro Imaging of Ministry of Education, School of Life Science and Technology, Xidian University, Xi’an, China

**Keywords:** Fluorescence imaging, Deep learning, Convolutional neural networks, Intraoperative pathology, Gliomas

## Abstract

**Purpose:**

Surgery is the predominant treatment modality of human glioma but suffers difficulty on clearly identifying tumor boundaries in clinic. Conventional practice involves neurosurgeon’s visual evaluation and intraoperative histological examination of dissected tissues using frozen section, which is time-consuming and complex. The aim of this study was to develop fluorescent imaging coupled with artificial intelligence technique to quickly and accurately determine glioma in real-time during surgery.

**Methods:**

Glioma patients (*N* = 23) were enrolled and injected with indocyanine green for fluorescence image–guided surgery. Tissue samples (*N* = 1874) were harvested from surgery of these patients, and the second near-infrared window (NIR-II, 1000–1700 nm) fluorescence images were obtained. Deep convolutional neural networks (CNNs) combined with NIR-II fluorescence imaging (named as FL-CNN) were explored to automatically provide pathological diagnosis of glioma in situ in real-time during patient surgery. The pathological examination results were used as the gold standard.

**Results:**

The developed FL-CNN achieved the area under the curve (AUC) of 0.945. Comparing to neurosurgeons’ judgment, with the same level of specificity >80%, FL-CNN achieved a much higher sensitivity (93.8% versus 82.0%, *P* < 0.001) with zero time overhead. Further experiments demonstrated that FL-CNN corrected >70% of the errors made by neurosurgeons. FL-CNN was also able to rapidly predict grade and Ki-67 level (AUC 0.810 and 0.625) of tumor specimens intraoperatively.

**Conclusion:**

Our study demonstrates that deep CNNs are better at capturing important information from fluorescence images than surgeons’ evaluation during patient surgery. FL-CNN is highly promising to provide pathological diagnosis intraoperatively and assist neurosurgeons to obtain maximum resection safely.

**Trial registration:**

ChiCTR ChiCTR2000029402. Registered 29 January 2020, retrospectively registered

**Supplementary Information:**

The online version contains supplementary material available at 10.1007/s00259-021-05326-y.

## Introduction

Glioma accounts for 75% of malignant primary brain tumor in adults [1]. Among these brain tumor patients, more than half are glioblastoma which is the most lethal glioma and have a median overall survival of only 14.6 months [2]. Although advanced therapies have been developed for glioma patients, neurological surgery remains the major treatment modality that plays an important role in improving survival.

At present, microsurgery under white light is the common method used in neurosurgery clinic, but it is difficult for neurosurgeons to clearly identify glioma boundary, resulting in tumor residual and early recurrence. Therefore, it is crucial to make quick and accurate diagnosis of dissected tissues during surgery. Intraoperative pathological examination of frozen tissue sections using hematoxylin and eosin (H&E) staining is a conventional and reliable diagnostic approach. But it usually costs a long time (at least 20–30 min) and requires complicated procedures to obtain pathological results [3]. Moreover, it is not practical to freeze ten or hundreds of samples during the operation, which limits its applications for intraoperative real-time diagnosis of tumor especially for multiple tissue samples.

In the past few years, artificial intelligence techniques such as several deep convolutional neural networks (CNNs) have been developed for classification of medical images and show high performance [4–10]. In coupling with conventional radiological imaging techniques such as magnetic resonance imaging (MRI), deep CNNs have also been used in the management and diagnosis of glioma to provide the grading [11] and genetic information [12], automate the pathological diagnosis [13, 14], and help determine prognosis and guide therapy [15]. However, all of these studies are mainly focused on preoperative planning and postoperative diagnosis and prediction.

Advances in fluorescence imaging [16–22] have brought real-time imaging-guided surgery to reality, which greatly increases complete resection rate in high-grade glioma. Indocyanine green (ICG) is a safe and economic near-infrared (NIR) fluorescence imaging agent that has been used in clinic to intraoperatively visualize glioma with high sensitivity and modest specificity [23]. We hypothesize that the application of CNNs to capture features from fluorescence imaging automatically has the potential to better provide accurate real-time visual information, and it helps neurosurgeons to distinguish tumor from non-tumor tissue during surgery.

**Fig. 1:**
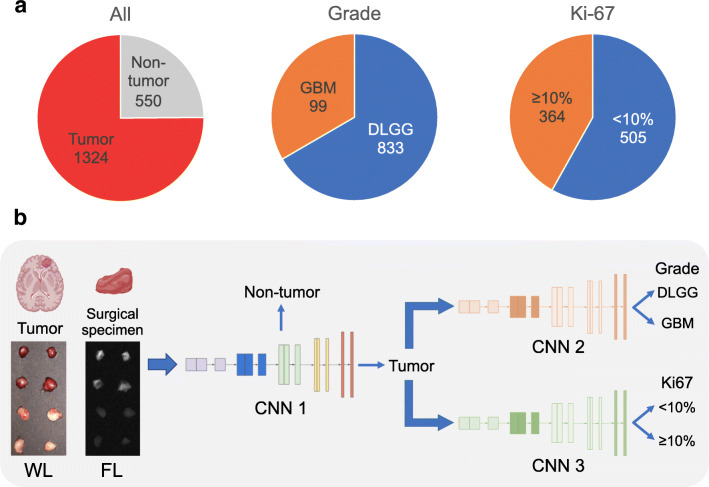
**a** Number of specimens per class with pathological examination results as the gold standard. Only tumor specimens had pathological results of tumor grade and Ki-67. **b** Specimens were resected by the guidance of NIR-II fluorescence imaging. Specimen images of WL and FL were obtained and fed into the CNNs for differentiation of tumor versus non-tumor. Images of tumor specimens were then fed into the CNNs for the classification of the grade and Ki-67 level. The prediction process by the models costed less than 1 s on CPU and can be faster on GPU; therefore, the time spent can be omitted

Herein, a dataset of NIR fluorescence (FL) and white-light (WL) images from 1874 specimens obtained from surgery of glioma patients was constructed (Fig. 1a). Then deep CNNs combined with the second NIR window (NIR-II, 1000–1700 nm) fluorescence imaging of ICG (named as FL-CNN) were developed to distinguish tumor versus non-tumor for all brain specimens, diffuse lower-grade glioma (DLGG, WHO II-III) versus glioblastoma multiforme (GBM, WHO IV), and low level (<10%) versus high level (≥10%, see the “Materials and methods” section) of Ki-67 for tumor specimens (Fig. 1b). Experiments were conducted on this dataset to validate the performance of the proposed FL-CNN. Moreover, the performance of FL-CNN was compared with WL-CNN (CNNs that take WL images as input), as well as to the results obtained from three neurosurgeons who read FL and WL images independently, using the pathological examination results as the gold standard.

## Materials and methods

### Study design

Our main research objective was to build CNNs (Fig. 1b, CNN 1, CNN 2, CNN 3) to distinguish tumor versus non-tumor for brain specimens, diffuse lower-grade glioma (DLGG) versus glioblastoma multiforme (GBM), and low level (<10%) versus high level (≥10%) of Ki-67 for tumor specimens based on their FL and WL images (FL-CNN and WL-CNN). A Ki-67 cutoff of 10% was chosen because it has been shown that few tumors with low proliferation rate were under the misclassification with it [24]. Therefore, we constructed a dataset consisting of WL and FL images from 1874 specimens from 23 patients with glioma. Of all specimens, using the pathological examination results as the gold standard, 1324 were tumor and 550 were non-tumor. Of all tumor specimens, 833 were DLGG and 99 were GBM, and 505 were with Ki-67 level < 10% and 364 were ≥ 10% (Fig. 1a). The data for three tasks were randomly split into a training set (70%) and a test set (30%) in patient level. More specifically, every patient was first assigned to the training set or the test set, and then all specimens from the patient were assigned to the corresponding set. The specimens from a given patient appeared only in the training set or the test set, but not in the two sets at the same time. The number of specimens per class in the test sets is provided (Supplementary Fig. S1). Because we split at the patient level and the numbers of specimens in the patients were uneven, the proportion of specimens in the test set is not exactly 30%. After training on the training set, we then evaluated and compared the performance of FL-CNN, WL-CNN, and neurosurgeons for the classification of tumor versus non-tumor on the test set. We further validated our CNNs in new tasks in the diagnosis of glioma, including the classification of the grade and Ki-67 level of tumor specimens, which is impossible for neurosurgeons during surgery.

### Study participants

Our dataset contained prospective, de-identified data from 23 patients enrolled from March 22, 2019, to April 22, 2020. Inclusion criteria included (1) male or female; (2) suspected as malignant glioma on preoperative contrast enhancement MRI; (3) voluntarily signed informed consent of surgical treatment and additional specimen beyond what was needed for routine clinical diagnosis; and (4) no contraindication of ICG. The study was approved by the Ethics Committee of Beijing Tiantan Hospital, Capital Medical University. All patients were given informed consent for their agreements. This study on patients with glioma was also explored in a clinical trial (ChiCTR2000029402) in China.

### Imaging system

The imaging unit of the intraoperative brain tumor imaging system consisted of a laser generator sub-system and a NIR-II and color image combined imaging instrument. The NIR-II imaging sub-system was composed of a cooled InGaAs charge-coupled device (CCD, NIRvana: 640, Teledyne Princeton Instruments) camera, a high-performance lens corrected for shortwave-wavelength infrared (SWIR) wavelength range (SWIRON 2.8/50, Schneider Kreuznach), and a spectral filter (FEL1000 longpass filter, Thorlabs) assembled with the lens through an adapter. The detector in NIR-II camera was Peltier-cooled to minimize thermally generated noise. The laser generator sub-system consisted of a laser, an optical fiber, and a collimator. The output wavelength of the laser was 808 nm, which was applied as the excitation light for NIR-II fluorescence imaging. The collimator converted the gathered light beam from the end of the fiber into a dispersed light spot with relatively homogeneous energy in it. The power of the laser was set as 50 mW/cm^2^. The NIR-II fluorescence image and the color image were acquired simultaneously. Besides the imaging unit, a controlling unit was also fitted in the system. This unit consisted of a laser distance–measuring instrument, a controlling handle, and a brunch of motors controlled by the handle. The motor enabled the imaging unit to rotate so that images of every side of the tumor cavity were acquired. The controlling unit was set for precise control of the working distance of the imaging unit and the reduction of touching the imaging unit, which might be above the operating field.

### Surgical protocol

ICG was injected intravenously to patients, radiological diagnosis of glioma, at a dose of 1 mg/kg (Dandong, Yichuang Pharmaceutical Co., Ltd., China), 48 h before anesthesia starting. The dosage and injection time were chosen by other trials on a few patients. Then, the NIR-II fluorescence imaging instrument was used to guide glioma resection intraoperatively. The white-light and NIR-II fluorescence images were acquired in the operating room. All patients’ tumor resections were performed by NJ who has more than 20 years’ experience of surgical treatment of gliomas. Specimens were obtained from tumor margin during the surgery. More specifically, the tumor surface was uniformly divided into 8 parts from the view of cross-section, and around 10 samples were taken from each part of the tumor-margin areas, resulting in a total of around 80 samples per patient (Supplementary Fig. S2). The resection of surgical specimens was guided by fluorescence imaging, which reduced the chance of sampling error. H&E and histochemically stained sections of the samples were used for diagnosis and assessment of the Ki-67 index. Tumor specimens were confirmed to be with enough tumor cell load by histology.

Three neurosurgeons (ZXG, SYH, JSC) who have similar surgical experiences and qualifications were watching operation at the time and independently evaluated brain specimens with access to intraoperative information such as anatomical location, vascularity, and perfusion. Because CNNs take every sample as input and predict its pathological diagnosis, for a fair comparison, we designed that three neurosurgeons independently read all of the samples and provided their per-sample evaluation results. All of the metrics of the neurosurgeons (readers) were also calculated based on these per-sample results. For grading, all specimens were also independently classified by three neuropathologists (GLL, JMW, SYT), according to the current World Health Organization Classification of Central Nervous System. In the case of a discrepancy, the 3 observers simultaneously reviewed the slides in order to achieve a consensus, and the corresponding immunohistochemical staining would be performed if necessary. To count Ki67, immunostained slides of tissue microarray (TMA) sections were scanned using a Leica Aperio AT2 scanner (at ×400 magnification), and the images were analyzed using a Leica Aperio ImageScope v12.3.0.5056. The algorithm was chosen according to the positive cellular location of each antibody. Cytoplasmic v2 algorithm was chosen for antibody positivity in cytoplasm, and nuclear v9 algorithm for nuclear positivity. The data of specimens diagnosed as gliomas were used for analysis. It should be noticed that a patient usually has both tumor and non-tumor specimens, and these tumor specimens are usually of different grades and Ki-67 levels. Therefore, it was certainly incorrect to apply the pathological results of a whole tumor to all samples from that tumor. All resected surgical specimens were sent for pathological examination. These pathological examination results of every specimen were used as the ground truth for model learning.

### Hardware and software

All experiments were conducted on Google Colab GPU Runtime (T4/P100 GPUs). PyTorch [25] (version 1.0+) was used for data loading, model building, and training. The final statistical analysis was performed in R (version 3.6.1) and Python (3.7), using pROC [26] (version 1.15.3) and sklearn [27] (0.21.3) for calculating receiver operating characteristic (ROC) statistics, sensitivity, specificity, accuracy, PPV, NPV, and *F*_1_ (*F*_1_ score is the harmonic mean of the PPV and sensitivity and ranges from 0 to 1). Figures and Tables were generated using matplotlib [28] (version 3.1.2) and Apache ECharts (version 4.6.0).

### Data preprocessing

Fluorescence images and white-light images of specimens were used as the only input of FL-CNN and WL-CNN. Fluorescence images produced by our fluorescence imaging system were not natural images like RGB color images or gray images and could not be used directly. The value of every pixel in the fluorescence image was the intensity of fluorescence signal, but not RGB value or gray value which ranged from 0 to 255. Therefore, pixel values of fluorescence images must be normalized nonlinearly to cope with traditional image processing pipeline. Tumor specimens produced strong fluorescence signal and non-tumor specimens produced weak fluorescence signal, so the normalized images of tumor specimens had larger pixel values and are brighter. It was noticed that these differences were only relative, but not absolute in the pixel values, because ambient light existed in the image and was likely to enhance the whole brightness. Randomly appeared white noises in the images might also influence these differences between tumor and non-tumor specimens. To solve these issues and make our CNNs more robust, heavy data augmentation was used to preprocess fluorescence images before CNN forwarding. Specifically, autocontrast and image denoising algorithms were applied firstly when needed to normalize the whole contrast of the image and remove noises. Then, random adjustment of brightness and contrast with a range of factor 1.0 was used to make CNNs more robust to absolute pixel value differences. Finally, the images were randomly flipped vertically, and patches were cropped from the images with a padding of 16. White-light images were natural RGB color images, so there was no need to do special preprocessing. Conventional data augmentation including random flip, rotate, and crop was used. The cropped patches from FL and WL images were sent to FL-CNN and WL-CNN, correspondingly. When testing, non-square images were zero-padded to meet the input requirements of CNNs.

### Problem formulation

Classification of tumor versus non-tumor for all brain specimens, and DLGG versus GBM and Ki-67 level < 10% versus ≥10% for tumor specimens were all binary classification problem. Probabilities of the presence of positive classes (tumor, GBM and ≥ 10%) were predicted by CNN 1, CNN 2, and CNN 3, respectively. To solve class imbalance, two coefficients were introduced to reweight the different parts of losses. The sum of the weighted binary cross entropy (BCE) losses across all images was given by the following equations:

1 $$ {L}_{\mathrm{BCE}}\left(y,\hat{y}\right)=-\sum \limits_{i=1}^m{\alpha}_P{y}_i\mathit{\ln}\left({\hat{y}}_i\right)+{\alpha}_N\left(1-{y}_i\right)\ln \left(1-{\hat{y}}_i\right) $$

2 $$ {\alpha}_P=\frac{\left|P\right|+\left|N\right|}{2\left|P\right|},{\alpha}_N=\frac{\left|P\right|+\left|N\right|}{2\left|N\right|} $$

where *y*_*i*_ indicates the probability of the presence of a positive class that a model predicted given *i*th input image, and ${\hat{y}}_i$ is the *i*th ground truth label. *α*_*P*_ and *α*_*N*_ are the balance coefficients of the positive and negative class, and ∣*P*∣ and ∣*N*∣ are the number of positive samples and negative samples for every task.

### Model development

CNN 1, CNN 2, and CNN 3 were developed for the classification of tumor versus non-tumor, DLGG versus GBM and < 10% and ≥ 10%. For FL images and WL images, different models called FL-CNN and WL-CNN were developed. Specimen images were firstly fed into CNN 1 to classify whether they were tumor or non-tumor. Then, images of tumor specimens were fed into CNN 2 and CNN 3 in parallel to predict their grade and Ki-67 level, respectively.

To solve these three binary classification problems on FL and WL images and obtain accurate and fast models, transfer learning was employed based on EfficientNet-B0 [29] which is one of state-of-the-art convolutional neural networks that achieves a good balance between performance and speed by carefully balancing network depth, width, and resolution. Compared with commonly used ResNets or Inception, EfficientNets can achieve the same level or better performance with an order of magnitude fewer parameters, which better meets the real-time requirements during the operation. The EfficientNet-B0 used here has only 5.3 M parameters and 0.39B FLOPS. For every task on FL or WL images, an EfficientNet-B0 was created and its parameters were initialized from the models pretrained on ImageNet. Then all layers except the last layer were used to extract features from images, and the last layer was replaced with a fully connected layer with a neuron for binary classification (tumor versus non-tumor, DLGG versus GBM, and < 10% versus ≥10%). The Xavier normal initializer [30] was used to initialize these fully connected layers. Dropout [31] was also applied before the last layer with a probability of 0.5 to avoid overfitting.

To better utilize the pretrained models and mitigate the issue of relatively few samples, there were 2 typical strategies for transfer learning to choose: (1) training a logistic regression classifier on the fixed feature extracted from the penultimate layer of the pretrained network, and (2) fine-tuning the entire pretrained network. The latter was found to be more effective for our problems. The optimization process used the Adam optimizer, with an initial learning rate of 0.001 and weight decay of 0.0001, using the weighted binary cross entropy loss function. The learning rate was decayed with a cosine annealing [32] every iteration with linear warmup. Early stopping was not used because of the utilization of cosine annealing strategy.

### Gradient-weighted class activation mapping

To gain insight into the representations learned by the CNN for classification, gradient-weighted class activation mapping (GCAM) was used for producing “visual explanations” for decisions, making the CNNs more transparent and explainable. FL and WL images of tumor and non-tumor specimens were randomly sampled, and their class-specific activation maps were presented, including colored GCAM, GCAM saliency, GCAM saliency × image, GCAM heatmap, and GCAM heatmap × image. Saliency × image/heatmap × image was generated by putting GCAM saliency/heatmap on the image, which made GCAM saliency/heatmap more recognizable**.** The last stage of the CNNs was used as the target layer and target classes were used to guide backpropagation and generate CAM.

### Statistical analysis

The ROC analysis and AUC were calculated to assess model differences for WL images and FL images on three tasks. All confidence intervals at 95% were computed based on 1000 iterations of the bootstrap method. Sensitivity, specificity, PPV, NPV, and *F*_1_ score of the FL-CNN for classification of tumor versus non-tumor were calculated at optimal decision thresholds with sensitivity >90%. Neurosurgeons’ performance was calculated based on the read results of the specimens in the test set. *F*_1_ score is complementary to the AUC and less sensitive than the AUC in cases of class imbalance. AUC, accuracy, sensitivity, specificity, and *F*_1_ score of the CNNs for classification of the grade and Ki-67 level were calculated at optimal decision thresholds that the point of the ROC curve was closest to the perfect (1,0) coordinate. A detailed transformation of predicted probability into decision using decision thresholds for three tasks on FL and WL images was provided (Supplementary Fig. S3). *P* values for comparisons were computed using a standard permutation test using 10,000 random resampling of the data.

## Results

### Classification performance of tumor versus non-tumor

**Fig. 2:**
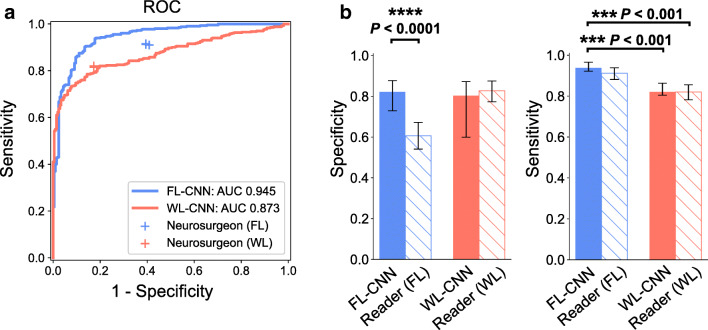
Diagnostic performance of the CNNs and neurosurgeons (readers). **a** Receiver operating characteristic (ROC) curves calculated for the CNNs and neurosurgeons on FL and WL images for the classification of tumor tissue versus non-tumor. The lines represent the ROC achieved by the CNNs. Individual neurosurgeon performance is indicated by crosses. Results of FL and WL are colored blue and orange, respectively. **b** Specificity and sensitivity of the CNNs and averaged individual neurosurgeons (readers) are plotted, using pathological examination results as the gold standard, and compared on FL and WL. The error bars represent the 95% confidence interval computed based on 1000 iterations of the bootstrap method. *P* values were computed using a two-sided permutation test with 10,000 random resampling of the data. All results were obtained on the test set (*N* = 608)

The performance of FL-CNN distinctly exceeded neurosurgeon’s performance in the classification of tumor tissue versus non-tumor (Fig. 2a). FL-CNN achieved an AUC of 0.945 (95% confidence interval (CI) 0.925–0.960), which performed much better than that of neurosurgeons (reader (FL) vs. reader (WL): sensitivity (0.912 vs. 0.820); specificity (0.606 vs. 0.827)). Furthermore, there was a significant difference between the performance of FL-CNN and WL-CNN (AUC of 0.873, 95% CI 0.843–0.900, *P* < 0.001). The results further demonstrated that FL-CNN not only significantly improved the specificity of diagnosis compared with neurosurgeons’ judgment based on FL images (Fig. 2b left, *P* < 0.0001) but also simultaneously achieved a higher sensitivity compared with neurosurgeons’ judgment based on WL images or WL-CNN (Fig. 2b right, WL images: *P* < 0.001; WL-CNN: *P* < 0.001). It was also noticed that, compared with neurosurgeons’ judgment on FL images, FL-CNN achieved significantly higher PPV (positive predictive value), NPV (negative predictive value), and *F*_1_ score (Table 1, all *P* < 0.05). While compared with neurosurgeons’ judgment on WL images and WL-CNN, FL-CNN achieved significantly higher NPV and *F*_1_ score (all *P* < 0.01). From error analysis, it was found that FL-CNN corrected most of the errors made by neurosurgeons (Supplementary Fig. S4). Over 2/3 of the all specimens were classified correctly by both FL-CNN and neurosurgeons. For the errors made by neurosurgeons, more than 70% of them (90/121, 86/117, 85/116 for FL images, 82/108, 83/108, 85/110 for WL images) were corrected by FL-CNN.

**Table 1:** Diagnostic performance of FL-CNN, WL-CNN and averaged individual neurosurgeons (reader) and the differences between FL-CNN and other three approaches, using pathological examination results as the gold standard

	Specificity	Sensitivity	PPV	NPV	*F*_1_
FL-CNN	0.822	0.938	0.910	0.872	0.924
	(0.729, 0.877)	(0.922, 0.966)	(0.866, 0.936)	(0.835, 0.925)	(0.900, 0.940)
Reader (FL)	0.606	0.912	0.817	0.783	0.862
	(0.541, 0.672)	(0.881, 0.938)	(0.778, 0.848)	(0.711, 0.843)	(0.834, 0.883)
	+0.216^*^	+0.025	+0.094^*^	+0.090^*^	+0.062^*^
	(0.125, 0.303)	(−0.038, 0.088)	(0.059, 0.129)	(0.008, 0.171)	(0.019, 0.104)
	*P <* 0.0001	*P* = 0.2106	*P* < 0.0001	*P* = 0.0159	*P* = 0.0017
Reader (WL)	0.827	0.820	0.901	0.705	0.859
	(0.773, 0.875)	(0.782, 0.855)	(0.868, 0.929)	(0.645, 0.762)	(0.832, 0.884)
	−0.005	+0.118^*^	+0.009	+0.168^*^	+0.065^*^
	(−0.096, 0.087)	(0.053, 0.185)	(−0.031, 0.049)	(0.094, 0.239)	(0.017, 0.113)
	*P* = 0.5216	*P* = 0.0003	*P* = 0.3231	*P* < 0.0001	*P* = 0.0042
WL-CNN	0.803	0.821	0.889	0.699	0.853
	(0.599, 0.872)	(0.804, 0.863)	(0.792, 0.926)	(0.644, 0.760)	(0.815, 0.878)
	+0.019	+0.118^*^	+0.021	+0.174^*^	+0.071^*^
	(−0.072, 0.111)	(0.050, 0.185)	(−0.019, 0.062)	(0.102, 0.247)	(0.022, 0.118)
	*P* = 0.3636	*P* = 0.0002	*P* = 0.1544	*P* < 0.0001	*P* = 0.0016

*Denotes that there are significant differences between these two results (*P* < 0.05)

### Feature visualization by GCAM

**Fig. 3:**
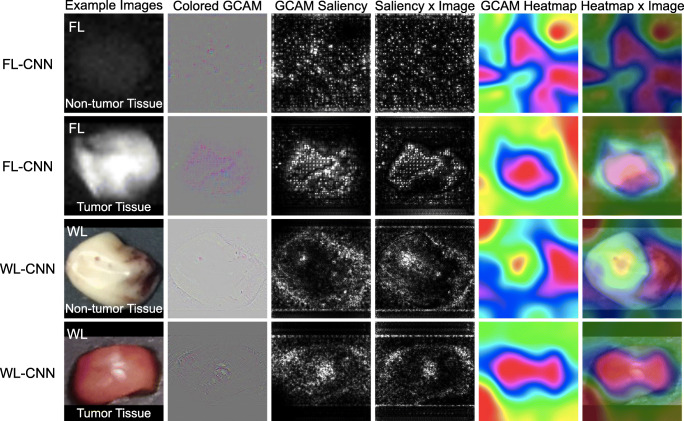
Gradient-weighted Class Activation Maps (GCAMs) visualize the learned feature representations for classification of tumor tissue versus non-tumor. FL/WL images of tumor/non-tumor tissues were randomly sampled. Colored GCAM, GCAM saliency, saliency × image, GCAM heatmap, and heatmap × image are shown. Saliency × image/heatmap × image was generated by putting GCAM saliency/heatmap on the image, which makes GCAM saliency/heatmap more recognizable. The target layer of GCAM was the last stage of the CNNs. Target classes were used to guide backpropagation and generate CAM. For preprocessing, non-square images were zero-padded to square, which led a black pad on the top/bottom or left/right

Gradient-weighted Class Activation Mapping (GCAM) produced colored GCAM, GCAM saliency, and GCAM heatmap which revealed recognizable features learned by CNNs for the classification of tumor tissue versus non-tumor (Fig. 3). For example, for FL-CNN, fluorescence signal of the non-tumor tissue was weak; therefore colored GCAM, GCAM saliency, and GCAM heatmap were sparse (first row). On the contrary, strong fluorescence signal of the tumor tissue produced dense-colored GCAM, GCAM saliency, and GCAM heatmap (second row). As for WL-CNN, there were differences between colored GCAM, GCAM saliency, and GCAM heatmap. Specifically, for the non-tumor tissue, colored GCAM and GCAM saliency concentrated more on the contours, but the GCAM heatmap put attention on the abnormal part (rightmost) of the non-tumor tissue where is rich in blood vessels. While for the tumor tissue, colored GCAM and GCAM saliency focused more on the contours and the center, but the GCAM heatmap cared the whole specimen.

### Classification performance of grade and Ki-67

**Fig. 4:**
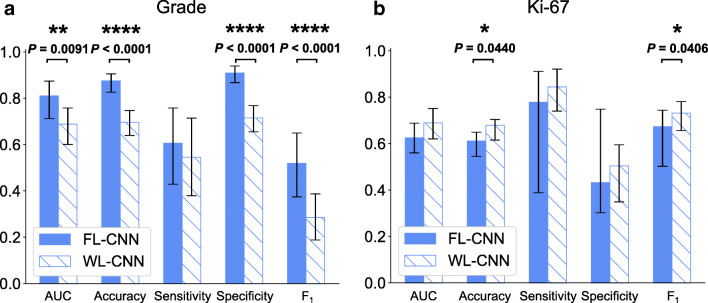
Performance of the CNNs for the classification of tumor grade and Ki-67 level. **a** Predicted grade of specimens are compared to the gold standard pathological examination results. AUC, accuracy, sensitivity, specificity, and *F*_1_ score of FL-CNN and WL-CNN are compared. **b** Predicted Ki-67 level of specimens is compared to the gold standard pathology results. AUC, accuracy, sensitivity, specificity, and *F*_1_ score of FL-CNN and WL-CNN are compared. All error bars represent the 95% confidence interval computed based on 1000 iterations of the bootstrap method. *N* = 296 for grade and *N* = 262 for Ki-67

It is well-known that neurosurgeons cannot obtain the grading and Ki-67 level information for the resected specimen during the operation. Importantly, here, we demonstrated that CNNs with strong learned features were generalizable to new tasks in the diagnosis of glioma, including the classification of the grade and Ki-67 level of tumor specimens (Fig. 4, Supplementary Fig. S5, and Table 2). FL-CNN and WL-CNN achieved AUC of 0.810 and 0.688 on the classification of grade (DLGG (grade II and grade III) vs. GBM (grade IV)), and 0.625 and 0.689 on the classification of Ki-67 level (< 10% vs. ≥ 10%), correspondingly. For the task of grade, FL-CNN achieved higher AUC, accuracy, sensitivity, specificity, and *F*_1_ score than that of WL-CNN. There were significant differences between their AUC, accuracy, specificity, and *F*_1_ score (all *P* < 0.01). While for the task of Ki-67 level, WL-CNN performed better in all of AUC, accuracy, sensitivity, specificity, and *F*_1_ score than that of FL-CNN. Among these metrics, WL-CNN achieved significantly higher accuracy (*P* = 0.0440) and *F*_1_ score (*P* = 0.0406). It was also found that those wrongly classified non-tumor specimens by FL-CNN in error analysis were mostly classified as DLGG or < 10%. The trend that FL-CNN showed better overall performance on the classification of tumor versus non-tumor was in agreement with the results on the task of grade, but not the task of Ki-67 level. In summary, the results on the classification of the grade and Ki-67 level highlighted the CNNs’ ability of learning and generalization.

**Table 2:** Performance of the proposed FL-CNN and WL-CNN on the classification of the grade and Ki-67 level of tumor specimens compared to the gold standard pathological examination results

		AUC	Accuracy	Sensitivity	Specificity	*F*_1_
Grade	FL-CNN	0.810	0.875	0.606	0.909	0.519
		(0.712, 0.874)	(0.826, 0.905)	(0.429, 0.758)	(0.867, 0.939)	(0.374, 0.650)
	WL-CNN	0.688	0.696	0.545	0.715	0.286
		(0.600, 0.758)	(0.639, 0.747)	(0.379, 0.714)	(0.655, 0.768)	(0.188, 0.387)
		+0.127^*^	+0.179^*^	+0.061	+0.194^*^	+0.234^*^
		(0.016, 0.235)	(0.108, 0.250)	(−0.152, 0.273)	(0.122, 0.266)	(0.110, 0.360)
		*P* = 0.0091	*P* < 0.0001	*P* = 0.2341	*P* < 0.0001	*P* < 0.0001
Ki-67	FL-CNN	0.625	0.611	0.778	0.433	0.673
		(0.560, 0.688)	(0.545, 0.649)	(0.388, 0.911)	(0.302, 0.748)	(0.502, 0.744)
	WL-CNN	0.689	0.679	0.844	0.504	0.731
		(0.620, 0.751)	(0.615, 0.729)	(0.740, 0.921)	(0.349, 0.595)	(0.656, 0.781)
		−0.063	−0.073^*^	−0.089	−0.056	−0.065^*^
		(−0.161, 0.034)	(−0.152, 0.007)	(−0.200, 0.022)	(−0.173, 0.063)	(−0.141, 0.007)
		*P* = 0.1013	*P* = 0.0440	*P* = 0.0594	*P* = 0.1839	*P* = 0.0406

*Denotes that there are significant differences between these two results (*P* < 0.05)

## Discussion

To the best of our knowledge, this study is the first demonstration of deep CNNs for predicting pathological diagnosis during fluorescence image–guided surgery. Previous works of deep learning on medicine are mainly concentrated on the fields of preoperative imaging and postoperative histopathology, aiming to automate analysis in an end-to-end way which saves time, resource, and labor. Unlike many of these works showing comparable performance to human experts, we have demonstrated that deep CNNs enable an approach to significantly improve accuracy of intraoperative diagnosis with zero time overhead, thus meeting the demand for fast and accurate tumor diagnosis during surgery.

Surgical resection is the main treatment regimen for glioma, which is associated with quality of life and survival [33]. With the updating operation concept for treatment, the maximum safe resection of glioma becomes highly important. Although more advanced imaging techniques have been applied intraoperatively, fast tumor diagnosis with high-throughput is still unavailable. To overcome this problem, FL-CNN has been developed and demonstrated to have the ability to efficiently distinguish tumor and non-tumor tissues during the neurosurgery. FL-CNN presents a high AUC of 0.945, sensitivity over 90%, and specificity over 80% on the classification of tumor versus non-tumor, which shows the potential to rapidly and precisely diagnose brain specimens and assist neurosurgeons to identify tumor boundary, thus protecting neurological functions during maximum tumor resection. Furthermore, our high-throughput method provides pathological diagnosis for even dozens or hundreds of specimens so that it can meet the demand of determining whether the tumor boundary requires further resection for neurosurgeons. In contrast to recent work using stimulated Raman histology and deep neural networks [3], we mainly focus on the specimen-level classification of tumor versus non-tumor and have conducted experiments on a larger dataset. Moreover, resection of surgical specimens in our method is guided by fluorescence imaging, which reduces the chances of sampling error. It is worth noting that our method using NIR-II fluorescence image not only guides tumor resection intraoperatively but also distinguishes tumor and non-tumor tissue especially in the boundary of the tumor.

The developed CNNs learn a non-linear mapping between fluorescence intensity distribution and pathological results, which is not like those defined a threshold for attributing a certain fluorescence level to be tumor or not. Therefore, they can differentiate tumor from non-tumor specimens better than neurosurgeons. Moreover, it can also provide effective information on grading and proliferation intraoperatively. These relatively objective evidences may assist surgeons in making individual resection decisions for each part of a tumor during operation, especially for these tumors which located in the functional area. If a specimen is suspected of being with high-grade and/or Ki67 level ≥ 10%, aggressive extent of resection should be taken into account for this site.

FL-CNN is established on NIR-II fluorescence imaging, which has a significant advantage over the first NIR window (NIR-I, 700–900 nm) in optical imaging characteristics, such as lower scattering and background signal [34], to produce a clear contrast between tumor and non-tumor tissue. Compared with clinically available 5-ALA fluorescence, NIR fluorescence typically has higher sensitivity and lower specificity (Supplementary Table S1). However, equipped with deep learning, our FL-CNN shows comparable specificity (0.822 vs. 0.811) than that of 5-ALA while keeping higher sensitivity (0.938 vs. 0.706). The experiment results have been confirmed by the gold standard of pathological examination, and the readers for diagnosis of specimens are with the same diagnostic experiences and qualification (Supplementary Table S2 and Supplementary Table S3), which ensures that the performance measurement of CNNs and neurosurgeons is precise, and the comparison between them is objective.

In the future, we plan to extend our CNNs to predict precise grading of tumors, in detail, from grade I to IV with a larger dataset. In addition, the proposed CNN-based approach also has the potential to predict significant genetic and molecular epidemiology of adult diffuse glioma, such as isocitrate dehydrogenase (IDH) mutation and 1p/19q chromosome co-deletion, which may provide more real-time intraoperative evidence for individual surgery strategies and early postoperative comprehensive treatment.

## Supplementary information

ESM 1 (PDF 745 kb)

## Data Availability

Data and materials are available for research purposes from the corresponding authors upon reasonable request.
