# Supplementary material for: Real-time intraoperative glioma diagnosis using fluorescence imaging and deep convolutional neural networks
Source: Eur J Nucl Med Mol Imaging. 2021 Apr 27;48(11):3482–92. doi: 10.1007/s00259-021-05326-y (PMC8440289; doi:10.1007/s00259-021-05326-y)
Supplement: Supplementary file 1 — (PDF 745 kb) [file 259_2021_5326_MOESM1_ESM.pdf]

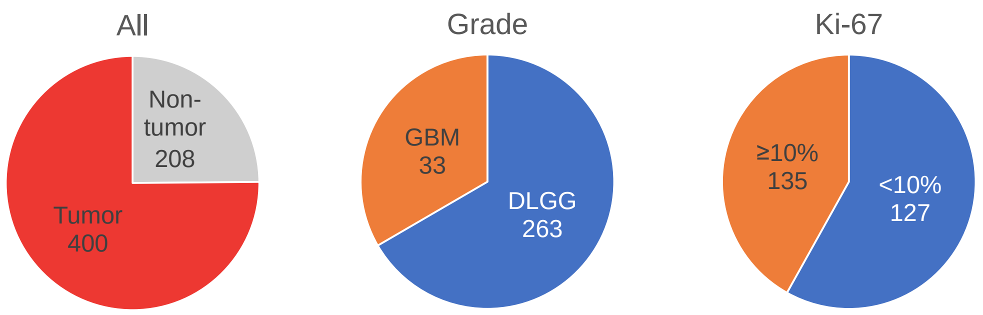

Supplementary Fig. S1 | Data distribution of test sets. Number of specimens per class of the test sets, with pathological examination results as the gold standard, for the task of classification of tumor versus non-tumor, DLGG versus GBM and Ki-67 level <10% versus ≥10%, correspondingly. Every test set was randomly sampled from the whole dataset while approximately keeping the class ratio as the original.

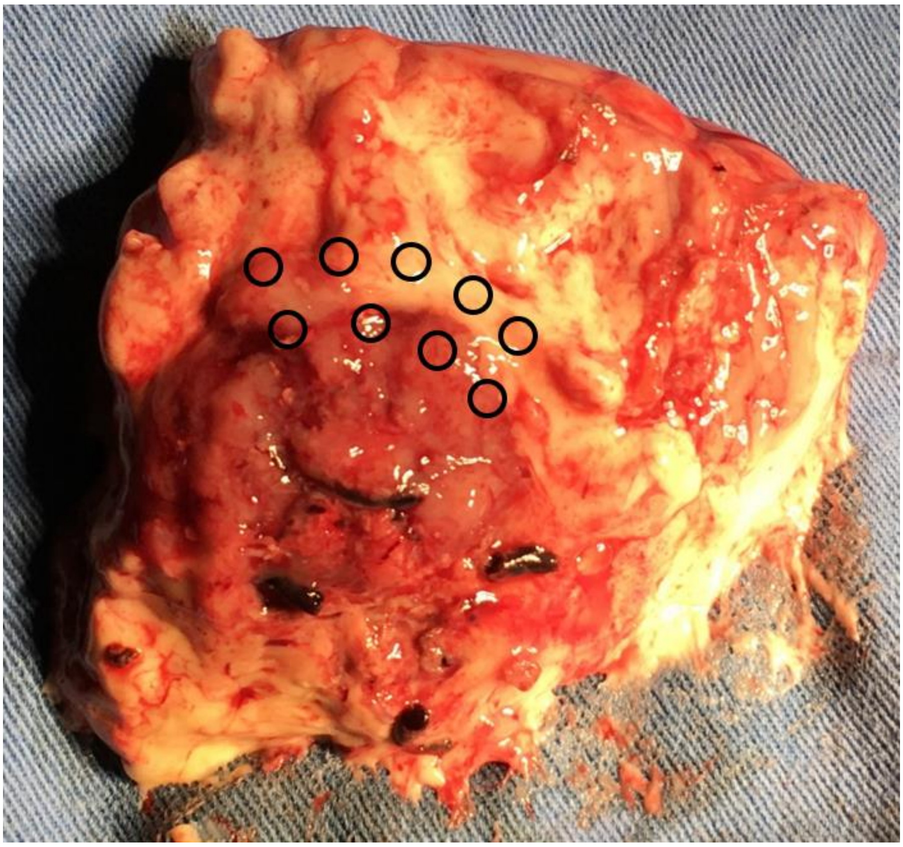

Supplementary Fig. S2 | Example for one of the 8 parts on tumor margin where surgical specimens were obtained from. The tumor surface was uniformly divided into 8 parts from the view of cross-section, and around 10 samples were taken from each part of the tumor-margin areas, resulting in a total of around 80 samples per patient.

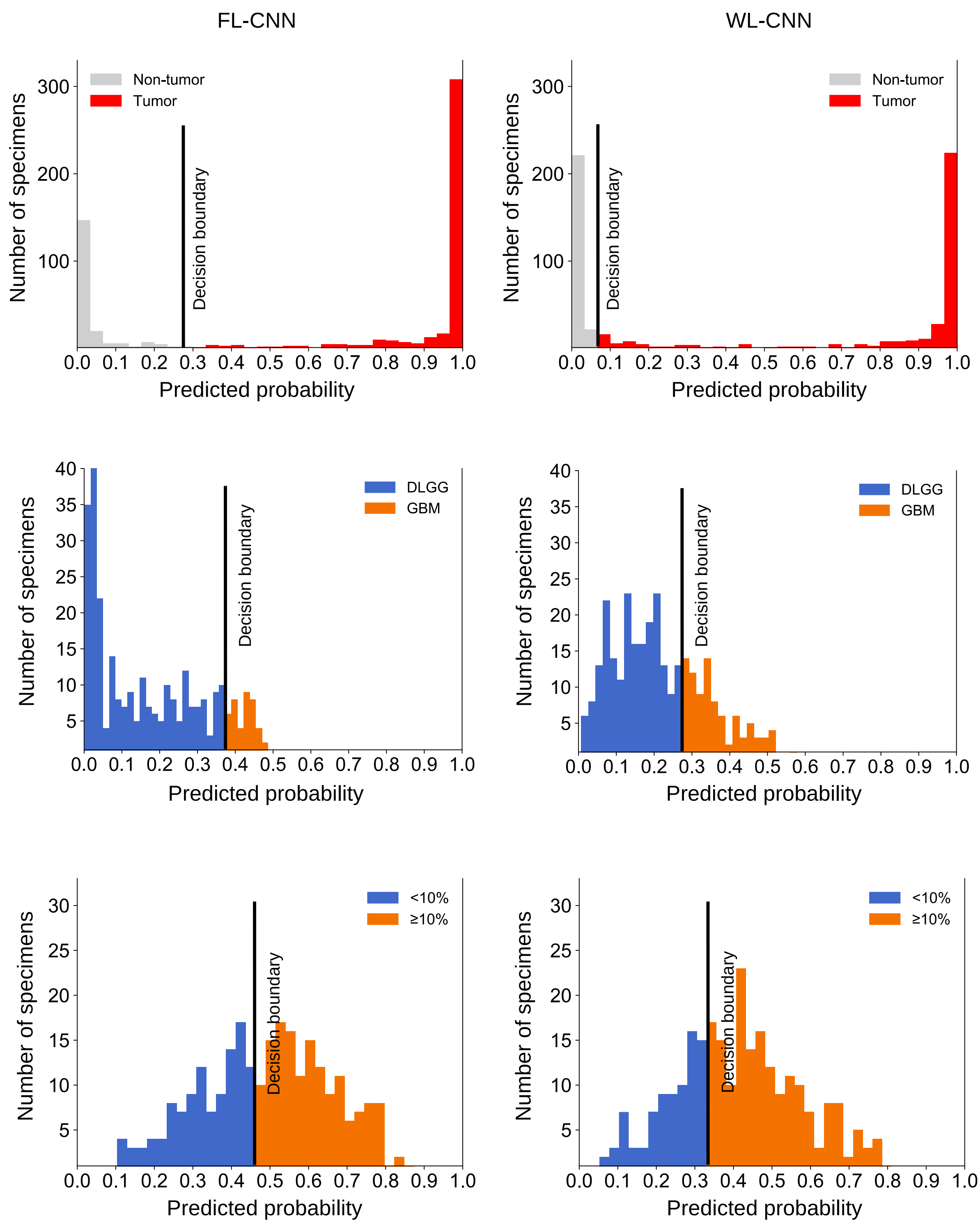

Supplementary Fig. S3 | Predicted probability distribution of CNNs on the classification of tumor versus non-tumor, and prediction of the grade and Ki-67 level of tumor specimens. Histogram plot of probabilities of the positive class (tumor, GBM, ≥10%) predicted by CNNs (FL-CNN and WL-CNN). Decision boundary is used to transform predicted probability into decision.

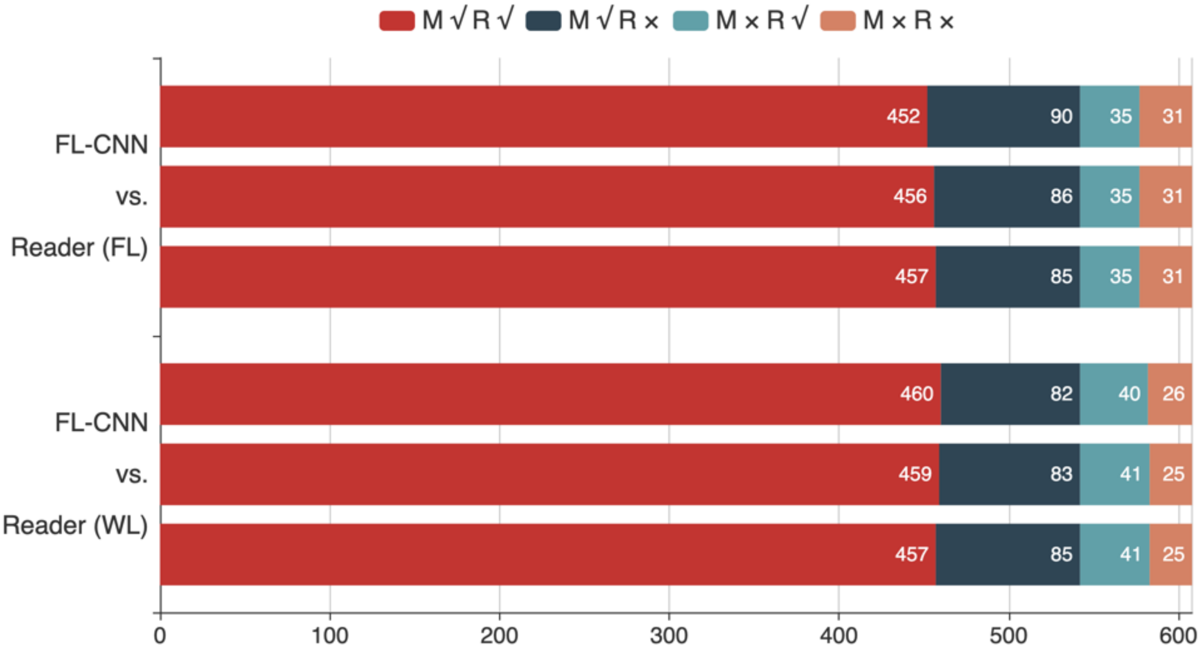

Supplementary Fig. S4 | Error analysis. Number of specimens classified right or wrong by FL-CNN and neurosurgeons. M represents the model (FL-CNN) and R represents neurosurgeons (readers). For example, M√R√ represents the number of specimens that both FL-CNN and neurosurgeons classified correctly; M√R× represents the number of specimens that FL-CNN classified correctly but neurosurgeons classified wrong, which means how many errors made by neurosurgeons were corrected by FL-CNN. M×R√ and M×R× follow the same convention. Three horizontal bars represent the comparison between three individual neurosurgeons and FL-CNN. All results were obtained on the test set (N=608).

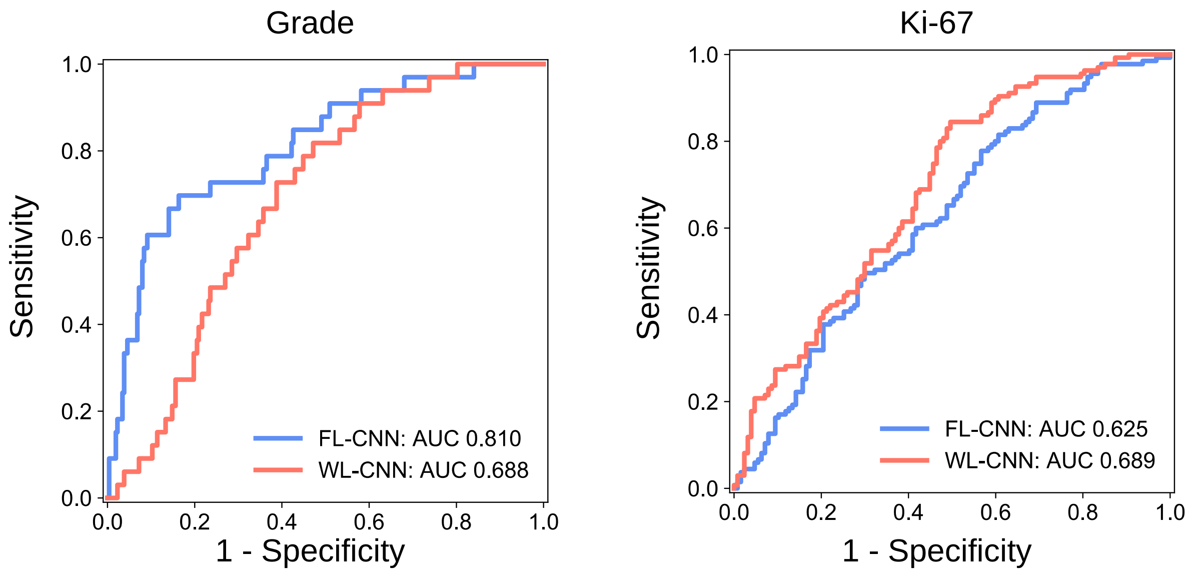

Supplementary Fig. S5 | ROC Analysis for the task of grade and Ki-67. Receiver operating characteristic (ROC) curves calculated for the CNNs on FL and WL images for the classification of DLGG versus GBM for grade, and <10% versus ≥10% for Ki-67 level. The blue lines represent the ROC achieved by the FL-CNN on FL images and red lines represent the ROC achieved by the WL-CNN on WL images.

Supplementary Table S1. Diagnostic performance of fluorescence imaging methods for brain tumors.

Reference	Method	Sensitivity	Specificity	PPV	NPV	Patient Size
John Y K L *et al.* *Neurosurgery.* 2016	ICG NIR-I	0.98	0.45	0.82	0.90	71
Stummer W *et al.* *J Neurosurg.* 2011	5-XLA	0.706	0.811	0.978	0.188	176
Stummer W *et al.* *Neurosurgery.* 2014	5-XLA	0.677	0.794	0.962	0.241	33
Acerbi F *et al.* *Clin Cancer Res* . 2018	Fluorescein	0.808	0.791	0.808	0.791	57

Supplementary Table S2. Inter-neurosurgeons and binary deep-learning method variability estimated with the Cohen's Kappa statistic, on the classification of tumor versus non-tumor for FL images.

	Neurosurgery 1	Neurosurgery 2	Neurosurgery 3	Consensus between 3 neurosurgeons	FL-CNN
Gold standard	0.531	0.548	0.551	0.548	0.770
Neurosurgery 1		0.983	0.979	0.983	0.497
Neurosurgery 2			0.996	1.000	0.514
Neurosurgery 3				0.996	0.517
Consensus between 3 neurosurgeons					0.514

Supplementary Table S3. Inter-neurosurgeons and binary deep-learning method variability estimated with the Cohen's Kappa statistic, on the classification of tumor versus non-tumor for WL images.

	Neurosurgery 1	Neurosurgery 2	Neurosurgery 3	Consensus between 3 neurosurgeons	WL-CNN
Gold standard	0.621	0.621	0.615	0.621	0.597
Neurosurgery 1		0.993	0.986	0.993	0.567
Neurosurgery 2			0.993	1.000	0.560
Neurosurgery 3				0.993	0.554
Consensus between 3 neurosurgeons					0.560
